# Alkaloids from In Vitro Cultured *Rhodophiala pratensis* Display Neuroprotective Effects in Murine Microglial Cell Models of Inflammation

**DOI:** 10.3390/plants15081186

**Published:** 2026-04-12

**Authors:** Diana Correa-Otero, Nandis Fiallos, Ángela Gómez-Mediavilla, Manuela G. López, Carlota Siguero-Gómez, Luis Bustamante, Julio Alarcón-Enos, Edgar Pastene-Navarrete

**Affiliations:** 1 Facultad de Ciencias, Universidad San Sebastián, Concepción 4030000, Chile; dianacorrea0114@gmail.com; 2 Laboratorio de Síntesis y Biotransformación de Productos Naturales, Departamento Ciencias Básicas, Universidad del Bio-Bio, Chillán 4081112, Chile; mahelymaravilla@gmail.com (N.F.); jualarcon@ubiobio.cl (J.A.-E.); 3 Departamento de Farmacología, Instituto Teófilo Hernando (ITH), Universidad Autónoma de Madrid, 28049 Madrid, Spain; angela.gomezm@uam.es (Á.G.-M.); manuela.garcia@uam.es (M.G.L.); csiguerogomez@gmail.com (C.S.-G.); 4 Departamento de Análisis Instrumental, Facultad de Farmacia, Universidad de Concepción, Concepción 4030000, Chile; lbustamante@udec.cl

**Keywords:** Amaryllidaceae alkaloids, culture in vitro, microglia, neuroprotection, nicotinic receptors, LPS

## Abstract

Neuroinflammation is determinant in the progression of neurodegenerative diseases. One of the main mechanisms underlying this process involves the persistent activation of glial cells. Persistent activation of glial cells induces proinflammatory transcription factors and the release of cytokines, chemokines, and reactive oxygen species that exacerbate cellular dysfunction. This neurotoxic environment promotes neuronal death, while the products of cellular damage feed back into glial activation, establishing a self-sustaining pathogenic cycle that drives neurodegeneration. Alkaloids present in Amaryllidaceae plants support the use of this resource in folk medicine, displaying potent effects as acetylcholinesterase inhibitors and allosteric modulators of nicotinic receptors (nAChR). In this study, a murine microglial cell (IMG) model of LPS-induced inflammation was used to evaluate the involvement of α7 and α4β2 nAChRs in glioprotection and neuroprotection of SH-SY5Y cells against 6-hydroxydopamine (OHDA). GC-MS analysis revealed differences in the alkaloid profile between in vitro cultures with fructose and wild-type *Rhodophiala pratensis*. Homolycorine-type, norbelladine-type and crinine-type alkaloids produced in vitro reduced LPS-induced inflammation (5 µg/mL), possibly via α7 and α4β2 nAChRs, and showed a protective effect against OHDA-induced oxidative stress (1–3 µg/mL) and inhibited AChE and BuChE (24–78 µg/mL).

## 1. Introduction

Neurodegenerative diseases constitute a heterogeneous group of diseases that affect the central nervous system and are characterized by progressive neuronal loss in specific areas of the brain [1]. Among neurodegenerative diseases, Alzheimer’s disease (AD) is the most prevalent and is estimated to account for 60 or 70% of dementia cases in the world. In the United States, approximately one in 10 people over the age of 64 have AD [2]. AD is characterized by extracellular plaque deposition of the amyloid beta peptide (Aβ) and abnormal intracellular neurofibrillary tangles of hyperphosphorylated microtubule-binding protein tau (τ), which in turn promote microglial activation [3]. Microglia are cells of the central nervous system (CNS) that function as elements of the immune system [4]. They are constantly examining the brain microenvironment, with the vital function of maintaining CNS homeostasis through the elimination of pathogens and cellular debris. In addition, they participate during the neuronal synapse process, controlling the number of neuronal precursor cells, among other functions [5,6]. However, in conditions of chronic inflammation, these cells can produce cytotoxic substances such as oxygen radicals, nitric oxide, proinflammatory cytokines like interleukin-1β (IL-1β), IL-6, and tumor necrosis factor-α (TNF-α) [7], contributing to neuroinflammation and neuronal loss [8,9,10].

Neuroinflammation not only triggers the onset of symptoms in Alzheimer’s disease (AD) but also constitutes a key factor in the pathogenesis of Parkinson’s disease (PD). In this context, the loss of dopaminergic neurons characteristic of PD is supported by abundant clinical and molecular evidence. Post-mortem studies in PD patients have demonstrated marked microglial activation, along with a high presence of various inflammatory mediators in the substantia nigra [11].

Given the important role of inflammatory processes in neurodegeneration, several studies have highlighted the involvement of nicotinic acetylcholine receptors (nAChRs) in the regulation of inflammation through the “cholinergic anti-inflammatory pathway”, which involves signaling between the vagus nerve and the brain, primarily mediated by the α7 nicotinic receptor [12].

This pathway is highly relevant for the systemic anti-inflammatory response in neuropathologies [12,13,14]. In addition, nAChr α4β2 is related to inflammatory pain and cognitive functions such as attention, learning and memory [15,16]. These receptors are expressed in neuronal and non-neuronal cells such as microglia, therefore, they are a crucial point of study in neurodegenerative diseases [10]. Since nAChR are involved in learning, memory, and neuroinflammation, orthosteric ligands have been developed to promote their activation. However, after prolonged exposure, it has been found that these drugs alter cholinergic transmission leading to desensitization of nAChR [17]. Therefore, the search for positive allosteric modulators (PAMs) that enhance the response induced by Ach, improving its efficacy and/or the probability of nAChR opening, is a very attractive strategy. These modulators have the advantage of having a low effect on the patterns of cholinergic brain activities [17,18,19,20].

Pharmacological studies have shown that a variety of selective agonists or positive allosteric modulators of the α7 nAChR improve the cognitive deficits, promote neuroprotection against neurotoxicants [21], and can mediate the neuroprotection caused by exposure to okadaic acid in human neuroblastoma cells (SH-SY5Y) through the Ca^2+^-independent JAK2/PI3K/Akt/GSK-3β signaling pathway [22].

Amaryllidaceae alkaloids (AAs) have well-known neuroprotective effects [23,24], thus, galantamine is a drug used and approved by the FDA for the symptomatic treatment of Alzheimer’s disease. It has a dual mechanism of action with effects on acetylcholine levels, acting as a reversible-competitive inhibitor of the enzyme acetylcholinesterase and as an allosteric modulator of nicotinic acetylcholine receptors [25,26,27,28]. Although inhibition of acetylcholinesterase (AChE) and butyrylcholinesterase (BuChE) are not related to neuroprotection [29,30], they are also related to AD. Indeed, one of AD’s hypotheses points to the decrease or absence of the neurotransmitter acetylcholine (ACh) due to the increased activity of the enzymes [31]. Furthermore, AChE has been shown to form a stable complex with senile plaque components through its peripheral anionic site. This site is located near the edge of the active site gorge of the enzyme and could be involved in accelerating the formation of β-amyloid (Aβ) fibrils [31,32,33].

Although, Amaryllidaceae alkaloids have great pharmacological properties, one of the biggest drawbacks in their use is obtaining them from wild plant material, of which the yield is around 1% or less. For this, the culture of plant cells and in vitro propagation allows us to obtain biomass without affecting the state of conservation of the plant, in a short time, and directing the production of a type of molecules according to the culture conditions [34,35,36]. The possibility of producing plant tissue or even whole plants in the laboratory not only reduces dependence on wild harvesting, but also directs the metabolic machinery towards the production of certain groups of alkaloids, reduces toxicity, or even promotes the synthesis of new alkaloids.

The production of carbohydrates (CHO) through photosynthesis not only sustains energy metabolism but also plays a central role in regulating plant growth and development by integrating endogenous and environmental signals [37]. Beyond their metabolic function, sugars act as signaling molecules that modulate complex regulatory networks, including conserved pathways such as SnRK1 and TOR, which coordinate cellular energy status with growth and stress responses [38,39]. In in vitro culture systems, the carbon source and concentration are critical factors influencing both development and secondary metabolism, as high CHO concentrations can induce osmotic stress and trigger metabolic adjustments that favor the accumulation of specialized compounds [40,41]. Furthermore, the nature of the sugar used can differentially modulate these responses. For example, in *Leucojum aestivum*, supplementation with fructose (30 g/L) significantly increases the production of alkaloids such as galantamine and lycorine [35].

Variations in the alkaloid production profile determine their pharmacological properties and, therefore, their potential therapeutic application.

In that way, the purpose of this study was to evaluate whether different alkaloids from *Rhodophiala pratensis*, (Amaryllidaceae) obtained in vitro under different culture conditions, have an anti-inflammatory and neuroprotective effect and if the observed effect is linked to nicotinic receptors. To achieve these aims, we quantified cell viability and nitrite production in mouse microglia models (IMG cells) exposed to lipopolysaccharide, in the presence or absence of selective and non-selective antagonists of the α7 and α4β2 nAChR. Furthermore, we evaluated the neuroprotective effect of alkaloids in a cell death model of human neuroblastoma (SY-SH5Y) cells exposed to 6-hydroxydopamine (OHDA), which is characterized by affecting dopaminergic neurons through the generation of reactive oxygen species, direct inhibition of complex I of the mitochondrial respiratory chain and release of inflammatory cytokines. These events also lead to glial activation [42] boosting neuroinflammation.

## 2. Results

### 2.1. Alkaloid Profile Analysis of Wild-Type Rhodophiala pratensis and Obtained in In Vitro Culture

Our research through GC-MS analysis allowed us to identify 22 alkaloids produced by wild-type *R. pratensis* (Table 1). On the contrary, only 7 and 5 alkaloids were produced in vitro culture supplemented with 30 g/L (30F) and 60 g/L (60F) of fructose, respectively (Table 1). Of the total compounds, we found that only trisphaeridine is produced both in 30F and in the wild plant. A previous study in different species of the genus *Rhodophiala* determined a total of 23 alkaloids. In this earlier work, the alkaloid distribution according to the oxidative phenol coupling was like that found in the present research [43].

**Figure 1:**
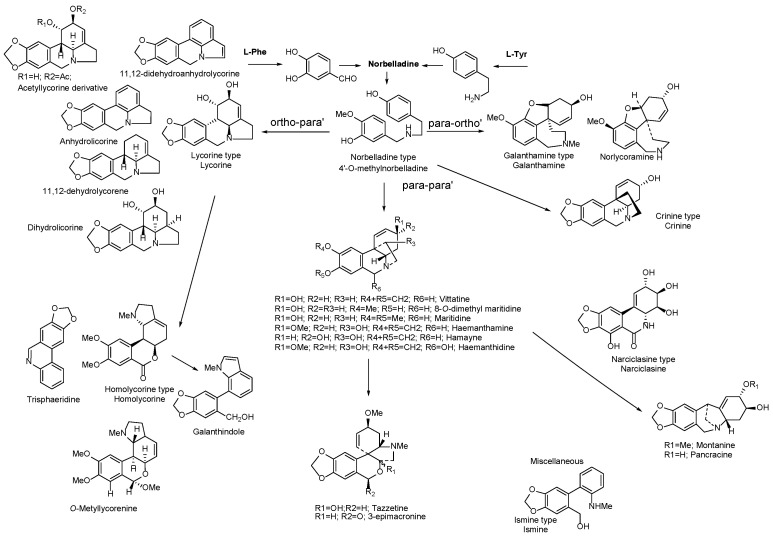
Scheme illustrating the phenol oxidative coupling pathways resulting in different Amaryllidaceae alkaloids.

Although the alkaloid profile is different for all samples, we found that the 30F and 60F culture conditions produce alkaloids by *para-para’* oxidative phenol coupling and with similar relative contents, thus we found 33.6% and 37.6% (Table 2), respectively. All alkaloids were identified following 2 criteria: (i) mass spectrum compared to literature (*m*/*z* fragments and relative intensity) and (ii) retention index. Figure 1 illustrates the different biosynthetic pathways that explain the origin of the main Amarillydaceae-type alkaloids identified on wild-type and in vitro cultured *R. pratensis* samples starting from 4′-O-methylnorbelladine as common precursor.

### 2.2. Cytotoxicity of R. pratensis Alkaloids on Mouse Microglia (IMG Cells)

To assess glioprotection and neuroprotection, we first studied the toxicity of the alkaloid extract obtained from wild plants and in vitro cultured plants.

**Figure 2:**
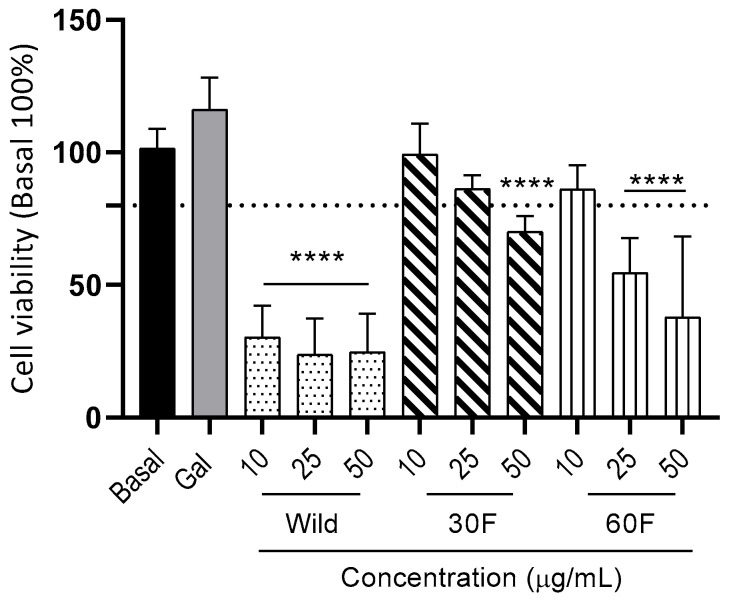
Toxicity of alkaloid extracts from wild *R. pratensis* and obtained in in vitro culture in microglial IMG cells. Glial murine cells were treated with increasing concentrations (10, 25, 50 µg/mL) of extract of wild plant alkaloids (Wild) and alkaloids produced in in vitro culture with 30 and 60 g/L of fructose (30F and 60F respectively). Galantamine (Gal) 5 µg/mL was used as a positive control. The data represent means ± SEM of triplicates of at least three different batches of cells. **** *p* < 0.0001 versus basal 0.4% dimethyl sulfoxide (DMSO) as vehicle.

We evaluated the alkaloid extract at the following concentrations. 10, 25, and 50 µg/mL. We found that the alkaloids from wild *R. pratensis* (Figure 2) displayed cytotoxicity above 65% at a concentration of 10 µg/mL, so these alkaloids were not used for protection experiments against LPS and OHDA.

The alkaloids obtained from 30F and 60F in vitro cultures at a concentration of 10 µg/mL presented toxicity of less than 20% (Figure 2), so they were selected as candidates for the subsequent experiments.

### 2.3. R. pratensis Alkaloids Protect Mouse Microglia When Exposed to LPS

**Figure 3:**
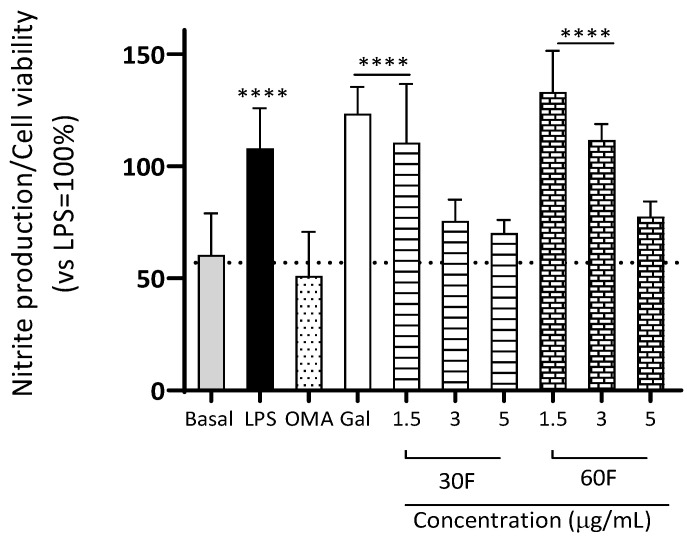
Nitrite production/cell viability ratio in IMG microglial cells exposed to 10 ng/mL LPS. Basal corresponds to cells treated with 0.4% dimethyl sulfoxide (DMSO) as vehicle; omaveloxolone (OMA, 7 µg/mL) was used as a positive control. Galantamine (Gal, 5 µg/mL) and alkaloid extracts (30F and 60F) produced with 30 and 60 g/L fructose culture condition, respectively, were also evaluated. Data represent the mean ± SEM of triplicates from at least three independent cell batches. **** *p* < 0.0001 vs. basal (0.4% DMSO vehicle).

Neuroinflammation is one of the most determining factors in various neurodegenerative pathologies [4]. For this reason, in this research, we used an in vitro model of microglia inflammation in which glial cells were exposed for 18 h to 10 ng/mL LPS. After this pro-inflammatory challenge, we determined the nitrites produced using the Griess method in relation to cell viability. As positive controls, we used galantamine and Omaveloxolone. This latter drug was recently approved by the FDA for the treatment of Friedreich ataxia [44]. Omaveloxone has anti-inflammatory and antioxidant activities via activation of Nrf2 and Nf-kB suppression, respectively. In our experiments, alkaloid extracts were tested at 5, 3, and 1.5 µg/mL (Figure 3). The results show that alkaloid extracts from 30F (3 and 5 µg/mL) and 60 F (5 µg/mL) treatments inhibit NO production in a concentration-dependent manner. These treatments did not present significant differences with omaveloxolone, our positive anti-inflammatory control. Considering the GC-MS composition, we found that the main compound in 30F is a homolycorine-type alkaloid (49.7%), while in 60F we found that the greater percentage corresponds to 4′-O-methylnorbelladine-type precursor (56.7%) alkaloids.

### 2.4. Nicotinic Receptors α7 and α4β2 Participate in the Defense of Microglia Exposed to LPS

**Figure 4:**
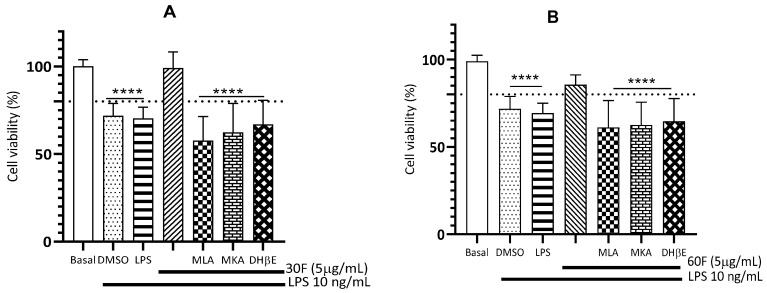
Involvement of α7 and α4β2 nAChRs in the protection of microglial exposed to LPS by alkaloid extracts obtained in vitro. (**A**) Extracts from 30F culture condition and (**B**) extracts from 60F culture condition. Basal corresponds to cells treated with 0.4% dimethyl sulfoxide (DMSO) as vehicle. Data represent the mean ± SEM of triplicates from at least three independent cell batches. **** *p* < 0.0001 vs. basal. (MLA, Methyllycaconitine (0.1 µM); MKA, mecamylamine (10 µM); DHβE, dihydro-β-erytroidine (1 µM).

As mentioned above, nAChRs are widely involved in neurodegenerative diseases. Through the modulation of these receptors and their multiple signal transduction cascades, the cholinergic anti-inflammatory pathway can be promoted [10,16]. To determine the participation of nAChRs in the observed protective effect, we quantified the cell viability of microglia exposed to LPS initially treated with selective antagonists for α7 and α4β2 such as MLA, DHβE, respectively, and non-selective antagonists such as MKA in the presence of 30F or 60F (Figure 4). None of the antagonists tested alone had a significant effect on LPS-induced cell death (Figure S1). Our results show that the protection that we noted above was reversed by preincubating the cells with nAChRs antagonists, indicating that nAChRs receptors (α7, α4β2, and others) are part of the anti-inflammatory mechanism displayed by alkaloid extracts produced in in vitro culture (Figure 4A,B).

### 2.5. Neuroprotection Assay Against Death SH-SY5Y Cells Induced by 6-OHDA

**Figure 5:**
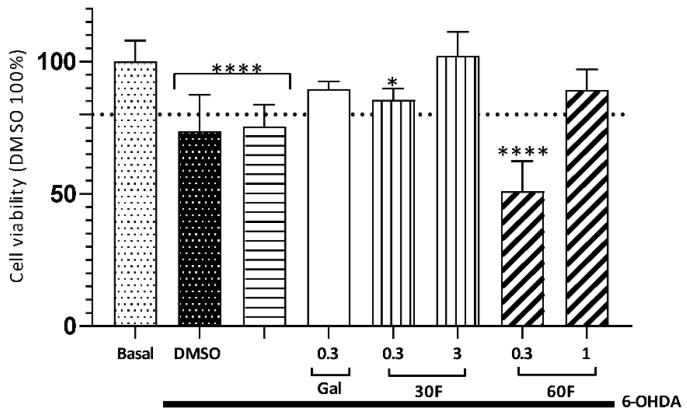
Protective effect of the alkaloid extract of *Rhodophiala pratensis* produced in in vitro culture with 30 and 60 g/L fructose (30F, 60F) in SH-SY5Y cells exposed to 6-OHDA 7 uM. Basal corresponds to cells treated with 0.4% dimethyl sulfoxide (DMSO) as vehicle. DMSO corresponds to cells treated with 0.4% DMSO and 6-OHDA. Galantamine 0.3 µg/mL (Gal) was used as a positive control. The data represent means ± SEM of triplicates of at least three different batches of cells. * *p* < 0.015, **** *p* < 0.0001, versus basal.

The neuroprotective effect of alkaloid extracts on SH-SY5Y cells exposed to 6-OHDA is represented in Figure 5. The neurotoxin significantly reduced cell viability by up to 75%, while galantamine and the 30F and 60F fractions, at concentrations 0.3, 3.0, and 1.0 µg/mL, respectively, protected neuroblastoma cells with viability equal to or greater than 90%.

Galantamine was used as a positive control to evaluate the neuroprotection of SH-SY5Y cells against 6-hydroxydopamine (6-OHDA). Galantamine is a widely studied allosteric modulator of nicotinic receptors, with known and reported effects such as neuroprotection and reduction of oxidative damage [28,30,45,46].

### 2.6. AChE and BuChE Inhibitory Activity

The inhibition (IC_50_) of AChE and BuChE for the alkaloid extracts obtained from in vitro culture and the wild-type plants are presented in Table 3. Galantamine, used as a positive control, presented IC_50_ values for AChE and BuChE of 0.56 and 2.0 µg/mL, respectively, which is consistent with a previous study [43]. While galantamine demonstrated high selectivity for inhibiting AChE, alkaloid extracts obtained in in vitro culture presented greater selectivity for BuChE than for AChE, with an IC_50_ value for 30F of 48.1 and 79.6 µg/mL and for 60F of 24.1 and 31.1 µg/mL, respectively. Fraction 30F is composed mainly of *ortho-para’-* (58%) and *para-para’* (~33.6%)-type alkaloids (Table 2) and presents a weak inhibition upon cholinesterase enzymes (>30 µg/mL). In contrast, the 60F fraction is constituted by precursor alkaloids or norbelladine derivatives (~57%) showing mild inhibition on BuChE. This is consistent with Al Mamun and collaborators [47], who synthesized norbelladin-type structures and determined, through in vitro, in silico, and kinetic experiments, that these molecules have great selectivity to inhibit BuChE.

## 3. Discussion

AAs are biogenetically related and have not been found in any other plant family [47,48,49,50]. Norbelladine is the precursor that can undergo different reactions and intramolecular couplings, which give rise to a myriad of diverse nitrogen-containing structures. The couplings can be *ortho-para’*-type in which lycorine and homolycorine-type alkaloids are obtained; *para-para’* in which crinine, haemanthamine, montanine, tazettine, and narciclasine-type structures are obtained; and the *para-ortho’* coupling that gives rise to galantamine-type alkaloids (Figure 1). The application of in vitro plant tissue culture to obtain molecules with biological activity is increasingly used [51], and the case of alkaloids is no exception. Thus, the production of montanine in *Rhodophiala bifida* has been investigated [34], the accumulation of lycorine in *Hippeastrum goianum* [52] and, most recently, the change in the alkaloid profile of four species of Chilean Amaryllidaceae using phytohormones [36]. In contrast with our results, the wild plant produces 81.1% of *para-para’*-type alkaloids, so the carbon source and combination of auxin and cytokinin modulate the production of alkaloids. In this case specifically, its effect was directly on the intramolecular phenol-oxidative coupling (Table 1 and Table 2). This aspect of our study is of central importance since it would allow, under laboratory conditions, to direct the metabolism of these plants towards alkaloids that have been poorly studied or even new in a simple way. Therefore, a notable finding is that in our research, we obtained alkaloid extracts with galantamine, crinine, narciclasine, and methylnorbelladine derivatives [24,53], structures never reported in Chilean species and with important pharmacological activities such as acetylcholinesterase inhibitors, and modulators of inflammatory pathways.

In the proposed strategy, apart from using fructose as an energy source, we sought to generate osmotic stress conditions in the plant by changing the usual sources of carbon. Table 1 shows the plant’s response to the change in sugar in the nutrient medium, where the alkaloid profile is clearly modified qualitatively and quantitatively by two fructose treatments, particularly in the appearance of 4′-*O*-Methylnorbelladine and homolycorine and crinine derivatives. Although the use of such an experimental approach has been reported early, we think that modulating the biochemical machinery of Amarillydaceae plants using different sugars is still an underexplored strategy. Our results suggest that the modification of concentration and type of sugar in the medium has an impact on the productivity and type of alkaloid synthesized in different Amarillydaceae. For instance, elicitors such as LED lights, salt stress, temperature, melatonin and auxins, and other plant growth regulators have been frequently reported [54,55]. Although it has been seen for other plant species, the effect of other types of salts and heavy metals could influence the biosynthesis of alkaloids. However, in the Amarillydaceae, this phenomenon is poorly explored [56,57]. As sucrose is the sugar that is conventionally used in culture media to improve the biosynthesis of alkaloids such as galantamine [58], the results of our work suggest that the strategy of replacing with a different sugar would serve to obtain new alkaloids or known alkaloids that are normally found in very low concentrations. Such limitations prevent their comprehensive pharmacological characterization.

It is well-known that the degree of toxicity of Amaryllidaceae alkaloids is a major problem that limits their clinical application in neurodegenerative pathologies. For this reason, its safety was confirmed in the cellular model used in this study (Figure 2). The results of this test coincide with an earlier neuroprotection study where it was determined that at 6 µg/mL alkaloid extracts from this wild plant presented toxicity greater than 20% [24]. These results indicate that despite being the same species, plant tissue cultures allow us to direct the production of compounds towards different biological activities or improving safety issues. On the contrary, if these alkaloid extracts are to be used for other purposes, the right combination of stimulants and carbon sources would allow for the predominance of other groups of alkaloids. For instance, recent studies have shown that the in vitro culture of *Caliphuria tenera* Baker supplemented with naphthaleneacetic acid (0.2 mg/L) and sucrose (30 g/L) was suitable for producing lycorine-type alkaloids, with cytotoxic effects on the epithelial cell line of human lung carcinoma A549 [59]. In addition, carbon sources have been reported to have a significant influence on alkaloid production [41].

A study found that fructose supplementation influenced the alkaloid profile, and saturates such as ismine, which is not produced in wild *Leucojum aestivum* plants, were found [35].

In the results presented in Figure 2, the alkaloid extracts with lower cytotoxicity were effective in protecting microglial cells from LPS-induced inflammation similar to the drug omaveloxone. This is an indication that Amaryllidaceae alkaloids may be NOS inhibitors against inflammatory stimuli such as an endotoxin like LPS. To date, some of the pharmacological properties such as cytotoxic, antitumor, and viral agents of this type of Amaryllidaceae alkaloids are known [60]. However, there is a paucity of studies focused on the neuroprotective effect of these structures. In this regard, Park and coworkers [61], reported the anti-inflammatory potential by inhibiting the activation of the Nfκ-B complex and cyclooxygenases in THP-1 cells exposed to LPS, so these results represent a new line of research regarding anti-inflammatory and NO inhibitory mechanisms in cells that could be of potential interest for the treatment neuroinflammatory diseases involving glial activation.

Regarding the effects displayed by the alkaloid extracts and the role of nicotinic receptors it is important to highlight that fraction 30F has 2.1% galantamine. We can attribute the observed effect to the fact that galantamine is a well-known nicotinic modulator that can promote their allosteric sensitization [62]. However, galantamine alone did not reduce inflammation at a concentration of 5 ug/mL while alkaloid extract in 30F and 60 F samples did provide a powerful protective effect. Interestingly, 30F and 60F extracts contain 12.9 and 28.9% of crinine derivatives, respectively. Previously, it had been reported that these types of alkaloids identified in *Crinum yemense* were able to inhibit the activity of iNOS in LPS-activated macrophages [63]. Another type of alkaloid with iNOS inhibiting properties is narciclasine [64]. Interestingly, chemical analysis found 4.8% of a narciclasine derivative (see Table 1) with a fragmentation pattern similar to trisphaeridine in 30F. These results suggest that our alkaloid fractions from in vitro cultures protect mouse microglia against the strong inflammatory stimulus caused by LPS.

Cellular exposure to LPS has been studied using in vivo and in vitro models. The microglial response to this molecule is characterized by activation and the subsequent production of cytokines and different pro-inflammatory neurotoxic factors [65,66,67].

Nitric oxide (NO) is a small molecule, which diffuses easily and is highly reactive. Although nanomolar concentrations of NO are necessary to maintain homeostasis, in a pathological event, microglia can produce it in micromolar concentrations by increasing the expression levels of inducible nitric oxide synthase (iNOS). The excessive NO production exacerbates neuroinflammation, causing neuronal death and tissue damage [68,69]. In a previous study, it was demonstrated that different NO activation products cause β-amyloid nitrotyrosination, accelerating its aggregation and the subsequent hyperphosphorylation of τau protein [70]. Another study found that in a rat model of LPS-induced learning impairment, NO inhibition reduced serum levels of inflammatory mediators, oxidative stress, and improved cognitive deficits [71]. Several studies have reported that nitrogen compounds derived from cyclic amidines and isoquinolinamines can inhibit iNOS [72]. Although, in this study galantamine, at the concentration evaluated, did not inhibit NO production. Egea and collaborators [30] have reported that in rat hippocampal slices subjected to anoxia/reoxygenation, galantamine activates the nicotinic acetylcholine receptor (nAChR) and activates Jak2, resulting in the inhibition of NADPH oxidase (NOX) and the prevention of p65 translocation to the nucleus, thus inhibiting the induction of iNOS. The alkaloids identified in the present work have an isoquinoline structure like galantamine suggesting that this may be a plausible mechanism of action.

Our work focused on aspects related to the protection that these alkaloid extracts provide against a massive production of NO in a model of neuroinflammation induced by LPS, measuring the viability of glial cells. Due to the greater relevance of reactive nitrogen species in this type of pathology, we decided to include an assay of NO production in IMG cells [73]. In glia, LPS increases nitrosative stress because this agent can induce the expression of iNOS, through the canonical pathway of NFkB (Nos2 gene). In fact, as expected, Figure 2 illustrates how this effect is counteracted by the alkaloid extracts produced in plants obtained under conditions 30F and 60F. The importance of this pathway has been highlighted early on in studies where genetic ablation of iNOS in APP/PS1 mouse models of Alzheimer not only increases cell survival, but also reduces two hallmarks of Alzheimer’s disease, beta-amyloid plaques, and hyperphosphorylated tau protein [74].

Due to its structural similarity, 6-OHDA enters cells using catecholamine transporters. Various studies have shown that the neurotoxic effect of 6-OHDA is mediated by blocking complex I of the mitochondrial respiratory chain [75]. Furthermore, in the autoxidation process, 6-OHDA produces hydrogen peroxide, anion superoxide radical and hydroxyl radical, which leads to oxidative stress. Due to the damage that this toxin produces at the neuronal level, this model as an indicator of oxidative stress is frequently used to simulate neuronal damage in Parkinson’s disease [76,77,78]. An homolycorine-type alkaloid is the major compound in the 30F alkaloid extract (Table 1). A previous study demonstrated that homolycorine-type alkaloids have a neuroprotective effect in SH-SY5Y cells in conditions of hypoxia and oxidative damage [79]. This compound is possibly the one that could be providing the greatest protection as is shown in Figure 4. Additionally, this 30F fraction contains galantamine, which may also have a protective effect via nicotinic receptors, an effect that has been demonstrated by different authors [10,29,62,80,81].

Omorury et al. reported that pretreatment with crinine-like alkaloids, belonging to the Amaryllidaceae family, effectively protects SH-SY5Y cells against MPP^+^-induced neurotoxicity, a widely used model for simulating dopaminergic neuron death. This neuroprotective effect was associated with the inhibition of reactive oxygen species (ROS) generation, the prevention of ATP depletion, and the reduction of cell apoptosis [82].

In this context, the alkaloid extracts obtained from in vitro culture, designated 30F and 60F, contain approximately 12.9% and 28.9% crinine-type alkaloids (Table 1), respectively. This difference in composition could explain, at least in part, the potent neuroprotective effect observed. In fact, fraction 60F showed efficacy at lower concentrations than 30F (1 µg/mL), which is likely related to its higher relative content of this type of alkaloid.

In fact, a clue of this statement is shown in Figure 4, where the microglia cytoprotecting effect of the alkaloid extracts from 30F and 60F conditions against LPS challenge was completely abolished in presence of MLA, MKA, and DHbE used to block α7 and α4β2 nAChRs nicotinic receptors. The effects observed with this extract (30F) could also be due to the ability of alkaloids derived from narciclasine such as trisphaeridine to stabilize radicals and molecules that cause cellular oxidative damage such as H_2_O_2_ [83]. As mentioned in Section 2.3, norbelladine derivatives can have potent anti-inflammatory effects against toxins such as LPS, which is probably why we observed the neuroprotective effect with 60F.

AChE and BuChE have crucial roles in the hydrolysis of acetylcholine (ACh), an important neurotransmitter at the synapse. Although AChE is highly specific for ACh, previous studies have shown that BuChE can metabolize various substrates and when AChE is inhibited, BuChE is capable of rapidly hydrolyzing ACh [32,84]. During the early stages of AD, AChE has accelerated activity; however, with the progression of the disease, AChE decreases and BuChE increases [43,85]. Therefore, the changes observed in the activity and expression of BuChE and AChE in AD highlight the potential value of these enzymes as a therapeutic target. Although an inhibition of both enzymes by the 30F and 60F fractions is observed with greater selectivity upon BuChE (Table 3), this effect is not comparable to that of galantamine. In this regard, it is important to highlight that alkaloid extracts correspond to mixtures of them and, therefore, this could explain why we do not observe such a pronounced effect.

In conclusion, using in vitro cultures of *R. pratensis* tissues, we successfully modified the alkaloid profile by replacing the carbon source with fructose. Two concentrations of fructose, 30F and 60F, allowed cell metabolism to be reoriented mainly towards alkaloids generated by *para-para’* couplings. These alkaloid extracts were less cytotoxic and displayed anti-inflammatory effects through the nicotinic acetylcholine receptor (nAChR) in glia exposed to LPS. In addition, these alkaloids protected neurons against the toxin 6-OHDA, which generates oxidative stress and mitochondrial damage. Additionally, our compounds inhibited the enzymes AChE and BuChE, which can contribute to cholinergic improvement in AD Advances in the study of neurodegenerative diseases increasingly highlight the importance of homeostasis between glia and neurons and how various factors can trigger neuroinflammation and oxidative stress. Therefore, the alkaloid extracts obtained in this study may be valuable resources in the search for new multitarget drugs to treat neuropathologies.

## 4. Materials and Methods

### 4.1. Reagents

6-benzylaminopurine, naphthaleneacetic acid, AChE from *Electrophorus electricus*, BuChE from equine serum, 5,5′-dithiobis-(2-nitrobenzoic acid) (DTNB), acetylthiocholine iodide, Lipopolysaccharide (LPS) from *Escherichia coli* serotype *O26:B6*, OMA (omaveloxolone), Galantamine, were purchased from Sigma-Aldrich. Codeine (purity > 99%) was purchased from Sigma Aldrich (St. Louis, MO, USA).

3-(4,5-dimethylthiazol-2-yl)-2,5-diphenyltetrazolium bromide (MTT Cat. No M2128-1G), N-(1-naphthyl)ethylenediamine, diamino diphenyl sulfone were purchased from Sigma-Aldrich, DMSO (VWR Avantor Cat. No. 23500.297).

### 4.2. Plant Material and In Vitro Culture

Wild bulbs of *Rhodophiala pratensis* were collected in Hualpén, Bio-Bio Region, Chile. Identification of the plant species was carried out in the herbarium of the University of Concepción, and they were labeled with the voucher *C. Baeza* 4340 (CONC).

The bulbs were sterilized following a standardized protocol from our laboratory [36]. Explants obtained through tween scales were plated in a sterile complete medium containing Murashige and Skoog salts, as well as 100 mg/L myo-inositol, 0.1 mg/L thiamine, 5 mg/L pyridoxine, 0.5 mg/L nicotinic acid, 2 mg/L glycine, and 8 g/L agar [86]. Additionally, the medium was supplemented with 2.20 µM 6-benzylamonopurine (BAP) and 5.40 µM naphthaleneacetic acid (NAA). Fructose was used as a carbon source at concentrations of 30 g/L (30F) and 60 g/L (60F) [35]. The growth conditions were maintained at a temperature of 25 ± 1 °C, with a photoperiod of 12 h under cold light with an intensity of 40 µmol/(m^2^s), and a relative humidity of 80%.

### 4.3. Extraction and Purification of Alkaloid Extract

Plant material obtained from in vitro culture and wild collection was lyophilized before extraction. The dried samples were extracted with methanol at a 1:25 (*w*/*v*) ratio using an ultrasonic bath (42 kHz, 70 W; Branson Ultrasonic Corporation, Brookfield, CT, USA). Sonication was performed over ten cycles of 30 min each, with 5 min intervals between cycles. The bath temperature was maintained below 45 °C throughout the process. The extraction solvent was replaced every two cycles to ensure extraction efficiency. All resulting methanolic extracts were combined and subsequently evaporated to dryness under reduced pressure using a rotary evaporator.

Alkaloid extract purification was carried out following the procedure reported by Cortés et al. [87], with minor modifications. The dried methanolic extract was dissolved in H_2_SO_4_ (2%) and subjected to three successive liquid–liquid extractions with ethyl acetate to eliminate pigments. The resulting aqueous phase was adjusted to pH 10 by the addition of NH_4_OH (25%) in order to yield free-base alkaloids. Hence, the alkaloid extracts were subsequently obtained by liquid–liquid extractions using chloroform.

The chloroform phases were evaporated to dryness under reduced pressure using a rotary evaporator and subsequently stored at 20 °C in Eppendorf tubes protected from light for further GC-MS analysis and biological assays.

### 4.4. Sample Preparation

Stock solutions of alkaloid extracts were prepared by dissolving 1 mg of dried extracts in 1 mL of DMSO and stored in the dark at 5 °C for subsequent use in cell culture experiments. All dilutions for cell-based assays (10, 25, 50 ug/mL) were prepared from the stock solutions (1 mg/mL), ensuring that the final DMSO concentration in each well did not exceed 0.4%.

For GC–MS chemical analysis, 4 mg of the alkaloid extract was dissolved in 4 mL of methanol. The solution was then sonicated for 30 s and stored in the dark at 5 °C until further analysis.

### 4.5. Identification and Quantification of Alkaloids by GC–MS

Analysis was carried out using gas chromatography-mass spectrometry (GC-MS) following a standardized method previously published in our laboratory [88]. In total, 1 μL of solution of alkaloid extract (4 mg/mL) was injected into an Agilent 7890 A GC (Agilent, Palo Alto, CA, USA) with multimodal injector and Agilent triple Quad 7000 GC/MS detector (analysis SCAN by quadrupole). The conditions were as follows: the oven was programmed from 100 to 180 °C at a speed of 15 °C/min, then from 180 to 300 °C at 5 °C/min, and held at 300 °C for 10 min.; the injection temperature was 250 °C. Helium was used as the carrier gas at a flow rate of 0.8 mL/min, and the separation was performed on an HP-5 MS capillary column (30 m × 0.250 mm × 0.25 μm, Agilent).

Codeine 50 μg/mL was used as internal standard to reduce variability in sample preparation. Alkaloids were identified based on their fragmentation patterns, and Kovats retention indices (RI) were calculated using the retention times of a homologous series of n-alkanes (C8–C12) and compared with values reported in the literature [24,34,43,89,90,91]. The area of all alkaloid peaks (usually base peak from the mass spectrum), in the samples were normalized to the area of the internal standard (*m*/*z* 299). Hence, through this approach (100% method), only the relative content of each compound was calculated, which is sufficient for comparison purposes between the same plant under different culture condition. The areas of all standardized peaks in the chromatogram were summed, and the ratio of each peak area to the total area was multiplied by 100. Chromatograms and mass spectra were analyzed with Agilent MassHunter Qual/Quant Workstation Software B.07.00. Agilent Technologies (https://www.agilent.com).

### 4.6. Culture of IMG Cell Line and Cell Viability Evaluation by the Colorimetric MTT Assay

The mouse microglial cell line (IMG), derived from the brain of 8-week-old C57BL/6J mice (Accession ID: CVCL_HC49 https://www.kerafast.com/item/1198/microglial-cell-line-img URL accessed on 05/02/2026), was kindly provided by Prof. Antonio Cuadrado from the School of Medicine, University Autonoma de Madrid. Spain.

Cells were maintained in high-glucose DMEM (Sigma, Cat. No. D65648), supplemented with 10% fetal bovine serum (Life Technologies Cat. No. A5256701), glutamine 2 mM (Life Technologies Cat. No. 25030024) and 1% Penicillin/Streptomycin (Merck Cat. No. P4333) in a humid atmosphere of 5% CO_2_ and 37 °C. IMG cells were sub-cultured in 96-well plates at a density of 13.500 cells/200 µL per well. For the cell viability assay, cells were incubated for 24 h with alkaloid extract at concentrations of 50, 20, and 10 µg/mL in growth medium. Cell viability was then determined using the previously described MTT method [92]. After aspiration of the cell media, the cells were incubated with 100 µL of a 0.075 mg/mL dilution of 3-(4,5-dimethylthiazol-2-yl)-2,5-diphenyltetrazolium bromide (MTT, Merck Cat. No M2128-1G) in DMEM without FBS for 45 min at 37 °C under an atmosphere of 5% CO_2_ and 95% RH. Subsequently, the solution was aspirated, and the crystals of formazan were resuspended in 100 µL of DMSO (VWR Avantor Cat. No. 23500.297). The absorbance was determined in a microplate reader at 570 nm (SPECTROstar Nano, BMG LABTECH, Ortenberg, Germany) after shaking the plate twice. The experiments were carried out in triplicate with cells from 3 different batches. Galantamine was used as positive control.

### 4.7. Anti-Inflammatory Activity Measured by the Griess Assay

IMG cells were sub-cultured in a 96-well plate at a cell density of 11,500 cells/200 µL per well. Pre-treatment of the cells was carried out for 24 h with crude alkaloids prepared at concentrations 5, 3, and 1.5 µg/mL in growth medium. Thereafter, cells were co-treated for 18 h with crude alkaloids and 10 ng/mL LPS. In this step, FBS levels were decreased to 1%. The inflammatory response of the cells was analyzed by measuring nitrite production by means of the Griess assay, as previously described [93]. Briefly, 70 µL of cell medium were transferred to another 96-well plate in the same order and mixed with equal volumes of the Griess reagents: first, 35 µL ice-cold dapsone (174 mg of diamino diphenyl sulfone +8.28 mL of 30% HCl, with water until 50 mL) were added followed by 40 µL NEDA (52 mg of N-(1-naphthyl)ethylenediamine in 50 mL of water). The mixture was incubated at room temperature protected from light for 5 min and light absorption was determined at 540 nm. Culture medium was used as negative control and relative nitrite concentration was determined and normalized against cell viability measured by the MTT method. Omaveloxolone (OMA) 7 ug/mL dissolved in 0.4% DMSO was used as a positive control.

### 4.8. Implication of nAChR in Protective Effects in Microglia

To evaluate whether the protective-anti-inflammatory activity of the alkaloids was mediated via nAChR, cells were pre-incubated with and without selective nicotinic antagonists such as methyllycaconitine (MLA 0.1 µM) for α7, dihydro-β-erythroid (DHβE 1 µM) for α4β2 and mecamylamine (MKA 10 µM) a non-selective antagonist [21,28]. Pretreatment was carried out 30 min before incubation with compounds or compounds + LPS. Finally, we determined cell viability by MTT.

### 4.9. Culture of SH-SY5Y Cell Line and Neuroprotection Against 6-Hydroxydopamine

Human neuroblastoma SH-SY5Y cells (ATCC^®^ CRL-2266, https://www.atcc.org/products/crl-2266) was kindly provided by Dr. Manuela García López. Cells were maintained in a 1:1 mixture of F-12 medium (HAM12) and minimal essential medium (MEM), supplemented with 15 non-essential amino acids, sodium pyruvate 1 mM, 10% inactivated fetal bovine serum (FBS), 100 U/mL penicillin and 100 µg/mL streptomycin (Invitrogen Reagents, Madrid, Spain). For neuroprotection assays, cells were plated in 96-well plates at a density of 50,000 cells/well and incubated for 24 h, until 80% confluency. A pretreatment was carried out for 24 h with the alkaloid extract at a concentration of 0.3–3 µg/mL. Thereafter, cells were co-treated for 24 h with the alkaloid extract and 7 µM 6-OHDA freshly prepared at the time of the experiment with MEM 1% FBS and 1.8 mM ascorbic acid. Viability was estimated by adding 100 µL of MTT (0.5 mg/mL) to DMEN without FBS and incubating for 45 min at 37 °C [94]. Galantamine (0.3 µm/mL) was used as a positive control.

### 4.10. AChE and BuChE Inhibition Assay

The AChE and BuChE inhibition assays were performed following a previously reported colorimetric methodology [95] with some modifications. AChE and BuChE were dissolved in 0.1 M PBS pH 7.2 in concentrations 4 U/mL and 8 U/mL respectively. The substrate consisted of 0.25 M acetylcholine iodide and 0.20 M DNTB (5,5-dithio-bis-(2-nitrobenzoic acid dissolved in PBS pH 7.2. The experiment was carried out on a 96-well plate, where 50 µL of crude alkaloids and 50 µL of enzyme were added. The mixture was homogenized with an orbital shaker at 90 rmp for 30 min at 37 °C. Subsequently, 100 µL of substrate was added, incubated for 15 min and orbital shaking at 37 °C. The hydrolysis control consisted of alkaloids, PBS and substrate. The positive control was galantamine. Reading was carried out at 405 nm in an Epoch 2 microplate reader spectrophotometer.

### 4.11. Statistical Analysis

Statistical analysis graphs and figures were performed using the software package GraphPad Prism version 5.0 (GraphPad software, La Jolla, CA, USA). All the experiments were conducted in triplicate. Means ± SDs are reported as an average of three independent assays. One-way ANOVA and the post hoc Dunnett’s test (95% confidence) were applied to address the significant differences compared to the control.

## Figures and Tables

**Table 1:** Alkaloid composition of extracts from wild-type *Rhodophiala pratensis* plants and specimens obtained through in vitro culture with 30 and 60 g/L fructose.

Compound	RI *	*m*/*z* (Relative Intensity %)	Relative Content (%)		
			W.T	30F	60F
Ismine	2274	**257 (30)**, 238 (100), 225 (6), 211 (7), 196 (10), 180 (9), 168 (9), 154 (5)	0.9	-	-
Trisphaeridine	2279	**223 (100)**, 222 (38), 167 (10), 165 (11), 164 (16), 138 (30), 111 (37)	0.9	15.9	-
4′-*O*-Methylnorbelladine derivative	2284	166 (36), 137 (100), 122 (8), 94 (6)	-	6.3	51.6
11-12-dehydrolycorene	2360	**253 (50)**, 252 (100), 224 (13), 166 (12), 152 (8), 139 (11)	0.1	-	-
Narciclasine-type	2388	**281 (1),** 259 (2), 250 (2), 225 (2), 157 (1), 147 (18), 129 (100), 112 (23)	-	4.8	-
Galantamine	2406	**287 (77)**, 286 (100), 244 (30), 230 (17), 216 (53), 174 (59), 128 (18), 115 (36)	-	2.1	-
Norlycoramine	2461	**274 (100)**, 202 (10), 188 (12), 178 (5)	0.3	-	-
Vittatine	2464	272 (13), **271 (82)**, 252 (8), 199 (100), 187 (91), 173 (32), 115 (42)	6.0	-	-
Homolycorine-type alkaloid	2476	**281 (2),** 252 (2), 207 (17), 191 (5), 179 (2), 164 (3), 125 (14), 117 (4), 110 (15), 109 (100)	0.5	-	-
Anhydrolycorine	2494	**251 (43),** 250 (100), 220 (2), 192 (14), 191 (13), 165 (4), 124 (19)	1.4	-	-
Galanthindole	2500	**281 (100)**, 264 (14), 263 (18), 262 (22), 252 (16), 204 (12), 191 (21), 132 (27), 107 (27)	5.0	-	-
8-O-Demethylmaritidine	2511	**273 (60)**, 230 (21), 201 (100), 189 (62), 174 (20)	0.6	-	-
Marithidine	2512	**287 (50)**, 270 (8), 268 (5), 258 (7), 244 (28), 215 (100), 203 (56), 196 (7), 167 (5), 128 (25), 115 (28)	2.3	-	-
*O*-Methyllycorenine	2529	**331 (1)**, 300 (-), 221 (3), 191 (0.5), 110 (8), 109 (100), 108 (11), 94 (2), 82 (2), 42 (1)	3.5	-	-
Crinine-type alkaloid	2540	**284 (5)**, 270 (40), 207 (15), 149 (11), 148 (100), 135 (40)	-	-	8.6
Crinine-type alkaloid	2580	**284 (26),** 284 (26), 267 (2), 207 (4), 161 (14), 148 (100), 135 (30), 107 (3), 89 (10)	-	12.9	28.9
11,12-Didehydroanhydrolycorine	2602	**249 (59)**, 248 (100), 190 (29), 163 (11), 123 (18), 95 (53)	0.7	-	-
Montanine	2622	**301 (97),** 270 (100), 257 (45), 252 (28), 223 (41), 185 (57), 115 (50)	10.1	-	-
Haemanthamine	2640	**301 (11),** 272 (100), 257 (12), 240 (21), 225 (10), 211 (23), 181 (47), 153 (20)	45.5	-	-
Tazettine	2649	**331 (12),** 316 (7), 298 (12), 247 (100), 227 (11), 211 (12), 201 (20), 181 (17), 152 (13), 115 (23)	11.3	-	-
Unknown	2670	**299 (14),** 267 (1), 164 (38), 163 (7), 151 (2), 207 (14), 137 (100)	-	-	5.1
Pancracine	2687	**287 (100),** 270 (25), 243 (34), 223 (33), 199 (51), 185 (67), 115 (51)	0.3	-	-
Hamayne	2707	**287 (5),** 258 (100), 242 (9), 212 (11), 211 (16), 186 (22), 181 (23), 153 (12), 128 (21)	2.0	-	-
Haemanthidine	2718	**317 (100),** 284 (48), 233 (67), 211 (47), 201 (89), 199 (79), 181 (71), 173 (66), 115 (89), 56 (44)	<0.1	-	-
Lycorine	2744	**287 (16)**, 268 (14), 250 (8), 227 (68), 226 (100), 211 (5), 147 (15)	4.4	-	-
Dihydrolycorine	2789	**289 (15),** 288 (97), 272 (28), 254 (40), 214 (25), 200 (2), 187 (22), 162 (15), 147 (46)	2.1	-	-
3-Epimacronine	2810	**329 (11),** 314 (12), 245 (100), 244 (24), 201 (78), 70 (29)	1.5	-	-
Acetyllycorine derivative	2892	330 (100), 270 (65), 254 (5), 226 (2), 147 (39)	0.5	-	-
Homolycorine-type alkaloid	3050	327 (5), **281 (34),** 253 (20), 207 (100), 191 (17), 177 (11), 158 (26), 125 (7), 109 (18), 81 (48)	-	8.3	-
Homolycorine-type alkaloid	3190	399 (8), 331 (1), 314 (15), **281 (30)**, 255 (17), 207 (100), 178 (14), 149 (18), 125 (10), 109 (25), 82 (7), 96 (23)	-	49.7	5.8

* Retention index; WT: wild-type, M^+^: was highlighted in bold.

**Table 2:** Classification of alkaloids by type of phenol-oxidative coupling of wild *R. pratensis* and produced in in vitro culture.

Alkaloid-Type	Wild-Type		30 F		60F	
	R.C	Number Alkaloids	R.C	Number Alkaloids	R.C	Number Alkaloids
*ortho-para*’	17.7	8	58.0	2	5.8	1
*para-para*’	81.1	12	33.6	2	37.6	2
*para-ortho*’	0.3	1	2.1	1	-	-
Miscellaneous	0.9	1	-	-	-	-
Precursor	-	-	6.3	1	51.6	1
Unknown	-	-	-	-	5.1	1

R.C: relative content (%).

**Table 3:** Cholinesterase inhibitory activity of alkaloid extracts from *R. pratensis* wild-type and in vitro culture.

Sample	IC_50_ ± SEM (µg/mL)	
	AChE	BuChE
*R. pratensis* wild-type	31.1 ± 2.0 ^a^	16.2 ± 2.8 ^a^
30F	79.6 ± 1.5 ^a^	48.1 ± 2.6 ^a^
60F	31.1 ± 0.6 ^a^	24.1 ± 0.5 ^a^
Galantamine	0.56 ± 0.01	2.0 ± 0.03

Results are expressed as means ± the SEM of three experiments. ^a^ *p* < 0.0001 versus the positive control (galantamine).

## Data Availability

The original contributions presented in this study are included in the article/Supplementary Material. Further inquiries can be directed to the corresponding author(s).

## Supplementary Materials

The following supporting information can be downloaded at: https://www.mdpi.com/article/10.3390/plants15081186/s1, Figure S1. Nicotinic receptor antagonists do not affect the cell viability of microglia exposed to LPS. Basal corresponds to cells treated with 0.4% dimethyl sulfoxide (DMSO) as vehicle. Methyllycaconitine (MLA 0.1 µM), dihydro-β-erythroid (DHβE 1 µM) mekamilamine (MKA 10 µM). LPS 10 ng/mL. ** *p* > 0.001.

## Abbreviations

The following abbreviations are used in this manuscript:

AAs	Amaryllidaceae alkaloids
Aβ	Amyloid beta peptide
ACh	Acetylcholine
AChE	Acetylcholinesterase
AD	Alzheimer’s disease
BuChE	Butyrylcholinesterase
CNS	Central nervous system
nAChR	Nicotinic receptors
6-OHDA	6-hydroxydopamine
