# Supplementary material for: Alkaloids from In Vitro Cultured Rhodophiala pratensis Display Neuroprotective Effects in Murine Microglial Cell Models of Inflammation
Source: Plants (Basel). 2026 Apr 12;15(8):1186. doi: 10.3390/plants15081186 (PMC13120194; doi:10.3390/plants15081186)
Supplement: Supplementary file 1 [file plants-15-01186-s001.zip › plants-4124130-supplementary.pdf]

SUPPLEMENTARY FILES

Alkaloids from *In Vitro* Cultured *Rhodophiala pratensis* Display Neuroprotective Effects in Murine Microglial Cell Models of Inflammation

Diana Correa-Otero [1,2], Nandis Fiallos [2], Ángela Gómez-Mediavilla [3], Manuela G. López [3], Carlota Siguero-Gómez [3], Luis Bustamante [4], Julio Alarcón-Enos [2] and Edgar Pastene-Navarrete [2,*]

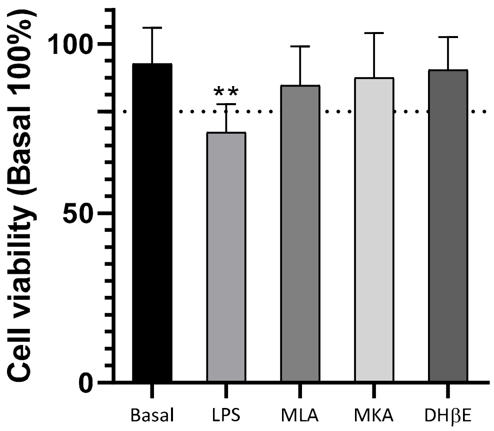

Figure S1. Nicotinic receptor antagonists do not affect the cell viability of microglia exposed to LPS. Basal corresponds to cells treated with 0.4% dimethyl sulfoxide (DMSO) as vehicle. methyllycaconitine (MLA 0.1 μM), dihydro-β-erythroid (DHβE 1 μM) mekamilamine (MKA 10 μM). LPS 10 ng/mL. ** p>0,001
